# Atypical Stress Cardiomyopathy and the Need for Multidisciplinary Care

**DOI:** 10.7759/cureus.61225

**Published:** 2024-05-28

**Authors:** Jack Jnani, Tafadzwa Mtisi, Tanzim Bhuiya, John Makaryus, Saaron Laighold

**Affiliations:** 1 Internal Medicine, North Shore University Hospital, Manhasset, USA; 2 Cardiology, North Shore University Hospital, Manhasset, USA; 3 Internal Medicine, Donald and Barbara Zucker School of Medicine at Hofstra/Northwell, Hempstead, USA; 4 Cardiology, Donald and Barbara Zucker School of Medicine at Hofstra/Northwell, Hempstead, USA

**Keywords:** transthoracic echocardiography (tte), left ventriculography, coronary artery angiography, cesarian section, reverse takotsubo cardiomyopathy

## Abstract

Reverse takotsubo cardiomyopathy is a rare variant of the classic stress-induced takotsubo cardiomyopathy. It is associated with transient left ventricular (LV) systolic dysfunction characterized by basal hypokinesis and apical hyperkinesis. We present a case of a 27-year-old woman who presented to an outside facility for a scheduled cesarean section and developed perioperative chest tightness, hypoxemia, and hypotension. Her electrocardiogram (ECG) showed sinus rhythm with marked ST segment depressions in leads V4-V6. High sensitivity troponin was elevated to 474 ng/L. Transthoracic echocardiography revealed an LV ejection fraction of 52% (Simpson's) with hypokinesis of the basal myocardial segments and hyperdynamic systolic function of the apical segments. Subsequent coronary angiography showed angiographically normal epicardial coronaries. Left ventriculography showed ballooning of the basal segments with apical hyperkinesis. She was subsequently diagnosed with reverse takotsubo cardiomyopathy and managed conservatively with beta-blockers. In this case, we highlight the need for collaboration between the cardiology and obstetric teams for tailored management strategies to ensure the well-being of both mother and baby.

## Introduction

Reverse takotsubo cardiomyopathy (rTTC) is a rare anatomical variant of takotsubo cardiomyopathy, a condition that is associated with transient left ventricular (LV) systolic dysfunction commonly in the presence of a physical or emotional stressor [1]. The mechanism is thought to be related to catecholamine excess in response to stress-causing myocardial dysfunction [2]. In rTTC, the typical apical ballooning seen in classic takotsubo cardiomyopathy is inverted with apical hyperkinesis and basal hypokinesis which can typically be seen by echocardiography [1]. The clinical presentation of rTTC can mimic that of acute coronary syndromes, but coronary angiography will usually reveal non-obstructed coronaries [3].

Although representing a minority of cases, rTTC presents a unique clinical picture. The prognosis of rTTC is generally favorable with a significant proportion of patients experiencing recovery of LV function within weeks to months [2]. However, complications such as decompensated heart failure, fatal arrhythmias, and thromboembolic events can occur though are less frequent than cases of classic takotsubo cardiomyopathy [1]. rTTC during the peri- or post-partum period may also pose unique clinical challenges. It can follow a significant stressor such as a cesarian delivery [1], as our case demonstrates. 

This article was previously presented as a meeting abstract at the 2023 American College of Cardiology Meeting on March 4, 2023.

## Case presentation

A 27-year-old female G3P3 presented to an outside hospital for a scheduled cesarean section. Approximately 10 minutes after receiving spinal anesthesia, she had new onset chest tightness, shortness of breath, headache with nausea, and some vomiting. Intraoperatively, she became hypotensive and hypoxic and required phenylephrine and decadron. She remained hypoxic in the recovery room and required supplemental oxygen. Her vitals were blood pressure 116/55 mmHg; heart rate 86 beats/minute, respiratory rate 18 breaths/minute; temperature 36.6℃; and oxygen saturation of 98% on 3L of nasal cannula oxygen. 

Differential diagnosis

The differential diagnosis for her chest pain with associated hypoxia and hypotension included stress cardiomyopathy, spontaneous coronary artery dissection, pulmonary embolism, peripartum cardiomyopathy, amniotic fluid embolism, acute coronary syndrome, and high spinal anesthesia. 

Investigations

**Figure 1:**
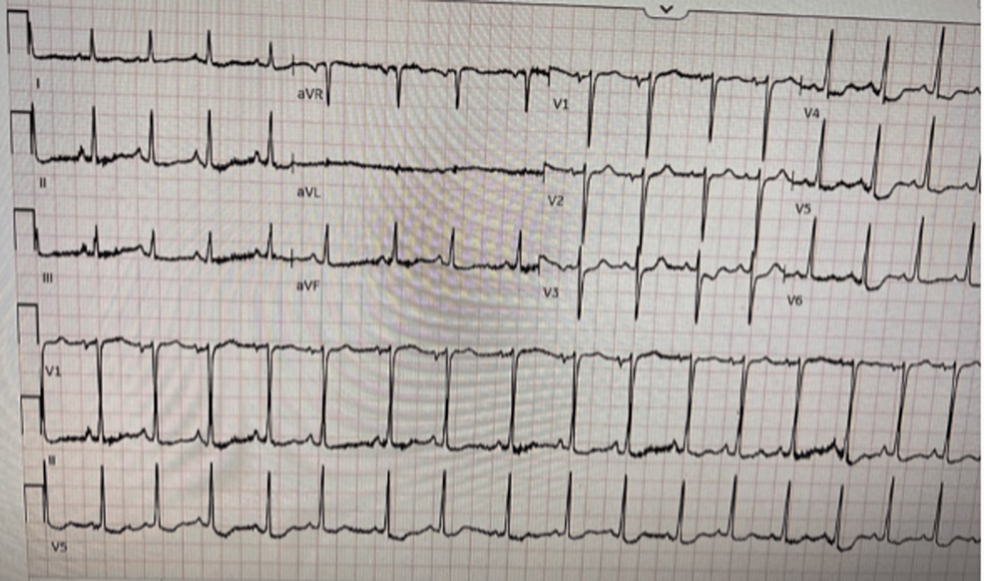
Electrocardiogram showing ST-segment depressions in leads V4-V6.

The patient was transferred to our tertiary institution for further investigation and management. Upon arrival, her high-sensitivity troponin-T enzyme was elevated to 474 ng/L with a brain natriuretic peptide level of 1247 pg/mL and a serum lactate of 2.7 mol/L. Her surface electrocardiogram showed sinus rhythm with marked ST-segment depressions in V4-V6 (Figure 1). Her chest x-ray demonstrated pulmonary edema. 

**Figure 2:**
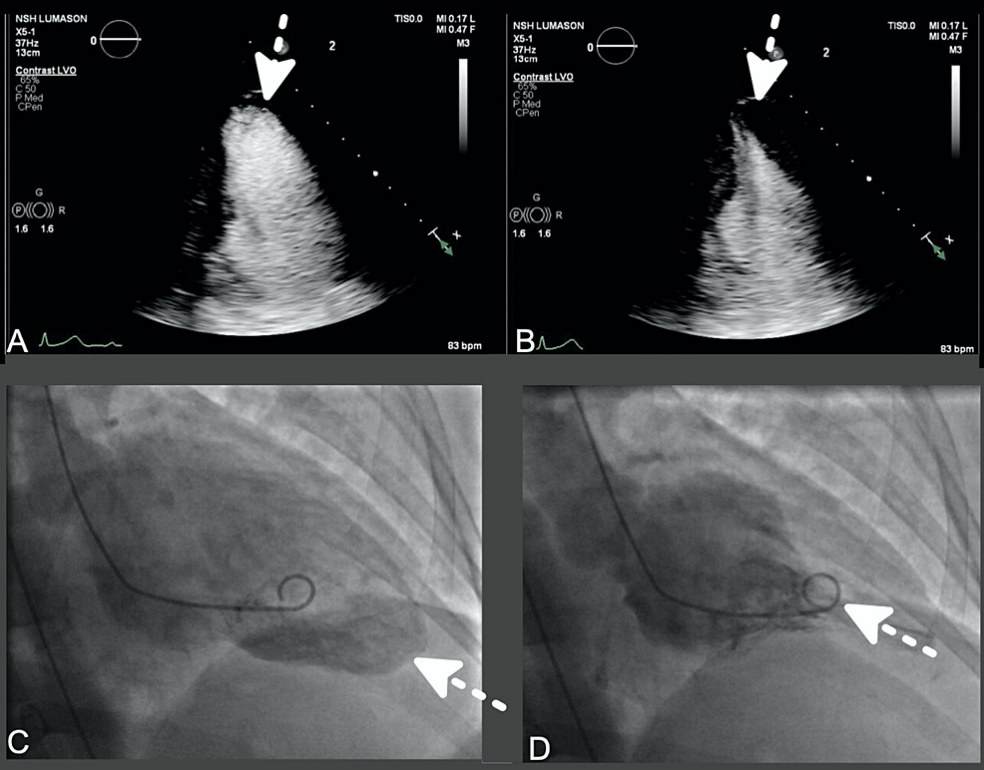
TTE four-chamber view with the use of definity echocardiography contrast at end-diastole (A) and end-systole (B). Left ventriculograms in end-diastole (C) and end-systole (D). Arrows point towards the cardiac apex. TTE: transthoracic echocardiogram

A transthoracic echocardiogram (TTE) revealed a LV ejection fraction of 52% (Simpson's) with hypokinesis of the basal myocardial segments and hyperdynamic systolic function of the apical segments (Figure 2A-2B). She subsequently underwent left heart catheterization, which revealed angiographically normal epicardial coronaries. Her left ventriculogram showed ballooning of the basal segments with apical hyperkinesis (Figure 2C-2D). 

Management

The patient was managed conservatively with beta-blockers. Since she was planning on breastfeeding her new child, the initiation of angiotensin-converting enzyme inhibitors (ACEi) was deferred. The obstetric team played a crucial role in her postpartum care and offered significant emotional support. The close collaboration between the obstetric, cardiology, and pediatric teams ensured the best possible outcomes for both mother and baby. 

## Discussion

Stress cardiomyopathy (takotsubo syndrome) is a well-recognized entity since its description in 1990 as an acute and transient LV dysfunction often following an emotional or physical stressful event [1]. It is classically associated with LV regional wall motion abnormalities extending beyond a single coronary artery distribution, typically with apical ballooning with basal hyperkinesis [1]. Typically, patients exhibit apical ballooning coupled with basal hyperkinesis [1]. In rTTC, or atypical stress cardiomyopathy, patients usually present with apical hyperkinesis and basal hypokinesis instead. 

Pathophysiological mechanisms

The mechanism of stress cardiomyopathy is not well understood. However, catecholamine excess may play a role. In response to physical or emotional stress, catecholamines are released which may cause myocardial damage directly or induce microvascular dysfunction or spasm [2]. There have been studies that demonstrated elevated levels of catecholamines in some patients with stress cardiomyopathy. In addition, coronary artery dysfunction may play a role. Left heart catheterization typically comes back negative for acute coronary obstruction. However, in some patients, it may demonstrate coronary artery spasm, especially with the provocative effects of acetylcholine [3]. Lastly, there may be some genetic basis for atypical takotsubo cardiomyopathy, as reported in familial cases of this disease with genetic polymorphisms in adrenergic receptors [4]. Similarly, rTTC hypothesized mechanisms include catecholamine excess, coronary artery spasm, microvascular impairment, and estrogen deficiency [5]. In addition, in the international registry of 1750 patients, 55.8% compared to 25.7% of patients had some history of psychiatric or neurologic disorder, demonstrating that this may be a very important risk factor for developing this disease [6]. 

Demographics

In a registry of 1750 patients with stress cardiomyopathy, about 89.8% were women and the mean age was 66.4 years [6]. While takotsubo cardiomyopathy presents most commonly in post-menopausal females, rTTC presents more commonly in younger females, such as in our patient. In a study on 60 patients with takotsubo cardiomyopathy, the mean age of rTTC was 36 years old compared to 62 years old in classical takotsubo [7].

In a large study of takotusbo cardiomyopathy of 97,650 patients, 86.9% were women, 91.8% were Caucasians, and 8.2% were African Americans [8]. Furthermore, in unadjusted analysis, African Americans had more cardiac arrests (3.8 vs. 2.9%), invasive mechanical ventilation (20.8% vs. 17.7%), acute kidney injuries (22% vs. 16.3%), and longer hospital stays (4.5 vs. 3.8 days) compared to Caucasian counterparts [8]. However, these findings were significantly attenuated after adjusting for comorbidities, socioeconomic status, and hospital location. African Americans had more in-hospital complications, but merely because of racial disparities in care [8]. Therefore, there is a significant need to address these racial disparities in the management of Takotsubo.

In an analysis of different types of stress cardiomyopathy, the apical type is the most common type (81.7% of patients), followed by the mid-ventricular type (14.6%), basal type, or rTTC (2.2%), focal type (1.5%), and global type (a very small minority of patients) [6].

Medical therapy

Stress cardiomyopathy is considered a transient disorder and is typically managed with supportive therapy. Removal of the physical or emotional stressor generally results in rapid resolution of symptoms [1]. However, some patients can develop acute complications such as heart failure and shock. A study by Templin et al. showed the rate of major adverse cardiac and cerebrovascular events was 9.9% per patient-year, and the rate of death was 5.6% per patient year in patients with takotsubo cardiomyopathy [6]. A 2018 study demonstrated that beta-blockers and ACE inhibitors did not affect the development, prognosis, or recurrence of the disease [9]. A more recent study in 2022 shows that patients receiving beta-blockers had a significantly lower risk for all-cause death (adjusted HR of 0.563) and non-cardiac death (adjusted HR of 0.525) compared to those not receiving beta-blockers [10]. 

The American College of Cardiology suggests that treatment requires inpatient care with cardiology services and is largely supportive until LV function returns, typically within 21 days [11]. Pulmonary congestion can be treated with diuretics and vasodilators in stable patients [11]. Additionally, the organization recommends the use of ACE inhibitors and beta blockers to reduce the workload of the heart and control hypertension [11]. In patients with unstable hemodynamics, echocardiography should be used to determine the presence of LV outflow tract obstruction. If one is present, inotropes should be avoided due to worsening obstruction [11]. Beta-blockers and intravenous fluids can be used in these cases. If an obstruction is not present, inotropes and vasopressors can be used if needed. Additionally, anticoagulation should be considered in patients with large areas of hypokinesis to prevent major cerebral or vascular events [12].

## Conclusions

rTTC commonly affects younger patients and is generally associated with less severe hemodynamic compromise. It can be precipitated by intense physical or emotional stress as our case demonstrated. Treatment usually entails conservative medical management with beta-blockers and ACEi or angiotensin receptor blockers (ARBs). Addressing racial and gender disparities in care delivery remains imperative. As our case demonstrates, clinical care delivery may be complicated necessitating multidisciplinary discussion between the cardiology and obstetric team to develop a treatment plan that takes the mother and baby's health into account. Close outpatient follow-up is also recommended for further management. 
